# Selective modulation of visual sensitivity during fixation

**DOI:** 10.1152/jn.00819.2017

**Published:** 2018-02-28

**Authors:** Chris Scholes, Paul V. McGraw, Neil W. Roach

**Affiliations:** Visual Neuroscience Group, School of Psychology, University of Nottingham, Nottingham, United Kingdom

**Keywords:** contrast sensitivity, facilitation, fixational saccades, microsaccades, suppression

## Abstract

During periods of steady fixation, we make small-amplitude ocular movements, termed microsaccades, at a rate of 1–2 every second. Early studies provided evidence that visual sensitivity is reduced during microsaccades—akin to the well-established suppression associated with larger saccades. However, the results of more recent work suggest that microsaccades may alter retinal input in a manner that enhances visual sensitivity to some stimuli. Here we parametrically varied the spatial frequency of a stimulus during a detection task and tracked contrast sensitivity as a function of time relative to microsaccades. Our data reveal two distinct modulations of sensitivity: suppression during the eye movement itself and facilitation after the eye has stopped moving. The magnitude of suppression and facilitation of visual sensitivity is related to the spatial content of the stimulus: suppression is greatest for low spatial frequencies, while sensitivity is enhanced most for stimuli of 1–2 cycles/°, spatial frequencies at which we are already most sensitive in the absence of eye movements. We present a model in which the tuning of suppression and facilitation is explained by delayed lateral inhibition between spatial frequency channels. Our data show that eye movements actively modulate visual sensitivity even during fixation: the detectability of images at different spatial scales can be increased or decreased depending on when the image occurs relative to a microsaccade.

**NEW & NOTEWORTHY** Given the frequency with which we make microsaccades during periods of fixation, it is vital that we understand how they affect visual processing. We demonstrate two selective modulations of contrast sensitivity that are time-locked to the occurrence of a microsaccade: suppression of low spatial frequencies during each eye movement and enhancement of higher spatial frequencies after the eye has stopped moving. These complementary changes may arise naturally because of sluggish gain control between spatial channels.

## INTRODUCTION

As we view the world, our eyes are never completely still. When we scan a scene, fixations on objects of interest are interspersed with fast, ballistic saccades, which serve to direct the most sensitive region of the retina (the fovea) toward these objects (Yarbus 1967). A great deal of research has been devoted to uncovering the effect that large saccades have on visual perception. However, relatively little is known about the effects on visual perception of the tiny eye movements that we make during fixation. To develop a complete understanding of how we visually sample the natural environment, it is thus imperative to characterize the effects that fixational eye movements have on visual input and the processes underlying visual stability.

Around the time of large voluntary saccades, behavioral studies have demonstrated marked decreases in visual sensitivity (Burr et al. 1994; Diamond et al. 2000; Knöll et al. 2011; Volkmann et al. 1978; Zuber and Stark 1966) and gross distortions of the perception of space (Cai et al. 1997; Honda 1989; Matin and Pearce 1965; Ross et al. 1997) and time (Morrone et al. 2005). Passive retinal processes, such as smear of the target across the retina during the saccade or masking of the target by pre- and postsaccadic spatial structure (Campbell and Wurtz 1978; Castet 2010; Dorr and Bex 2013; Macknik and Livingstone 1998), are likely to dominate the changes in sensitivity during natural viewing (Wurtz 2008). However, there is also evidence for an active process in which a corollary discharge or copy of the neural signal associated with a saccade is used to cancel out the effect of the eye movement. Diamond et al. (2000) demonstrated that saccadic suppression occurs even when smear and masking are controlled for and, critically, that it is absent when image motion consistent with a saccade is simulated via a rotating mirror. In their study, saccadic suppression began up to 50 ms before the saccade commenced, which, considered alongside physiological evidence for presaccadic modulation of neural activity in monkey middle temporal, medial superior temporal, and ventral intraparietal areas (Bremmer et al. 2009), suggests that there is a critical active extraretinal contribution to saccadic suppression. An important feature of saccadic suppression is its selectivity for the spatial frequency of the stimulus, with the largest effects occurring at lower spatial frequencies and little or no suppression at higher spatial frequencies (Volkmann et al. 1978).

During periods of fixation, our eyes drift slowly and every 500 ms or so we make small, involuntary microsaccades (Barlow 1952). Microsaccades are generally considered to be miniature versions of larger saccades: for example, they occur at a similar rate of 1–2 per second (Steinman et al. 1967, 1973), they exhibit the same relationship between peak velocity and amplitude (Zuber et al. 1965), they are followed by increased drift velocities (Chen and Hafed 2013) similar to the glissades observed after larger saccades (Bahill et al. 1978), and they are generated by the same neural circuitry in the superior colliculus (SC) (Hafed and Krauzlis 2012). Because of these similarities, and comparable changes in visual neural responses for saccades and microsaccades (Chen et al. 2015; Hafed et al. 2009; Hafed and Krauzlis 2010; McFarland et al. 2015), perceptual changes due to microsaccades are generally accepted to be qualitatively similar to the suppressive effects reported for larger saccades. In support, early studies showed that the probability of detecting broadband flashes was lower before and during microsaccades (Beeler 1967; Zuber and Stark 1966), and more recent physiological studies have demonstrated microsaccadic suppression of behavioral responses (Chen and Hafed 2017; Hass and Horwitz 2011).

Despite the evidence above, there have been suggestions that microsaccades (and fixational eye movements in general) may play special roles in vision. For example, it has been suggested that fixational eye movements may actually improve, rather than hinder, visual sensitivity. By considering how eye movements redistribute the spatiotemporal power of a stationary target, Rucci and colleagues have argued that ocular drift and microsaccades have a differential impact on the detection of grating stimuli. Drift selectively attenuates power at low spatial frequencies, making it easier to detect high-spatial frequency stimuli embedded in noise (Boi et al. 2017; Rucci et al. 2007). In contrast, power at low spatial frequencies is preserved during microsaccades, which should act to increase sensitivity relative to periods of drift (Mostofi et al. 2016). So, on one hand suppression during large saccades is strongest for low spatial frequencies, while on the other hand smaller saccades might act to improve sensitivity in the same frequency range. As a result, there is a need to examine the impact of microsaccades on visual sensitivity more closely.

We parametrically varied the spatial frequency of a stimulus during a detection task and collected thousands of trials to track sensitivity as a function of time relative to randomly occurring microsaccades. We show that spatial sensitivity is suppressed before and during microsaccades, predominantly at low spatial frequencies, much like that noted for larger saccades. When the influence of microsaccades is mapped over a sufficiently long time period, we find a significant facilitation of sensitivity, centered on spatial frequencies of 1 cycle/° and above. Surprisingly, enhancement of sensitivity occurs ∼100–200 ms after microsaccade onset, i.e., once the microsaccade has ended. We demonstrate that facilitation is unlikely to be due to postmicrosaccadic ocular drift. Instead, we present a model in which the spatial tuning of suppression and facilitation can be explained on the basis of a delayed normative gain operating between spatial frequency channels. Overall, our results demonstrate that microsaccades, which occur frequently during fixation, have measurable, and perhaps unique, perceptual consequences that cannot simply be inferred by extrapolating from current knowledge about larger saccades.

## MATERIALS AND METHODS

### 

#### Participants.

Data were collected from 15 individuals (9 men, 6 women; mean age = 25 yr, range = 20–47 yr), 11 of whom were inexperienced at performing visual psychophysics and were naive to the purpose of the experiment. All participants had normal or corrected-to-normal vision and provided written informed consent. The study was reviewed and approved by the institutional ethics committee and conducted in accordance with the Declaration of Helsinki at the time the data were collected (Version 6, 2008).

#### Stimulus materials and procedure.

**Fig. 1.**
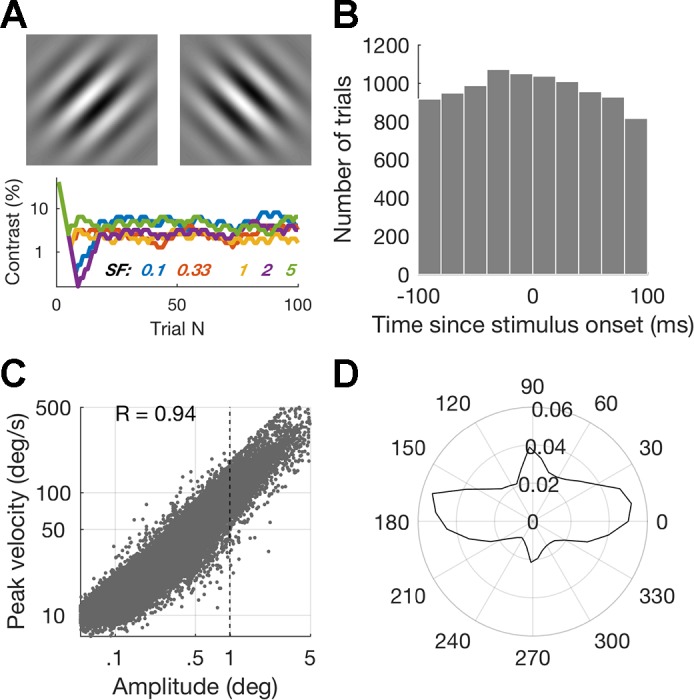
*A*: example of the stimuli used in the task (not to scale) and example staircases for 1 subject at each of the spatial frequencies (in cycles/°). *B*: no. of trials containing microsaccades, binned according to the interval from stimulus to microsaccade onset. *C*: microsaccade peak velocities show a fixed relationship with their amplitude. Dashed line indicates the cutoff amplitude of 1° used for subsequent analyses. *D*: directional distributions of microsaccades as a proportion of total no. of microsaccades: the majority of microsaccades were horizontal.

Participants sat in a dark room and were instructed to maintain fixation on a central white dot (0.08° diameter, Weber contrast 0.95). The head was secured with a chin and forehead rest. Stimuli were large Gabor patches presented centrally (standard deviation of 5°, 1 frame duration at 85 Hz). Carrier phase was randomized to prevent the buildup of a retinal afterimage, and orientation was randomly set to ±45° (see Fig. 1*A*). Participants were prompted to respond by an auditory tone synchronized to stimulus onset. To minimize the effect of expectation (Hafed et al. 2011; Pastukhov and Braun 2010) on microsaccade rates, the interval from participant response to the next stimulus onset was randomly selected for each trial from a uniform distribution (800–4,000 ms).

Stimuli were generated with PsychoPy (Peirce 2007, 2009) and displayed on a 20-in. CRT monitor (Iiyama Vision Master Pro 514; resolution = 1,024 × 768, viewing distance = 75 cm, background luminance = 45 cd/m^2^). The luminance response of the monitor was gamma-corrected, and 14-bit grayscale resolution was obtained with a Bits++ stimulus processor (CRS, Cambridge, UK). Stimulus contrast was adaptively varied for each of five interleaved 1-up 3-down staircases each with a different spatial frequency (0.1, 0.33, 1, 2, or 5 cycles/°). Subjects indicated the orientation of the Gabor (±45°) with the left and right arrow keys.

#### Eye movement analysis.

**Fig. 2.**
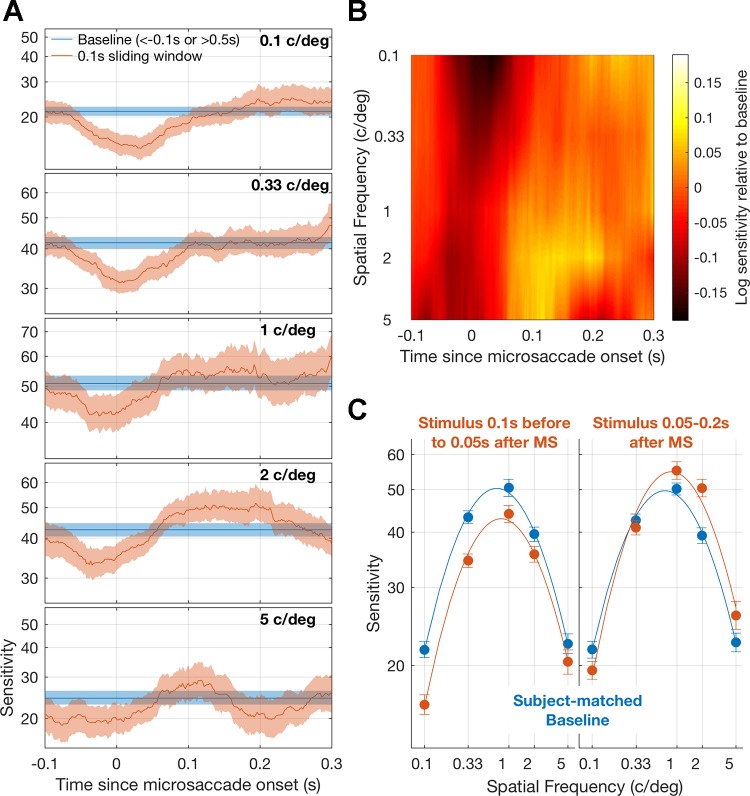
Visual sensitivity relative to microsaccade onset. *A*: sensitivity at each time point was calculated by binning trials in 100-ms time windows (moving in 2-ms steps) and fitting a logistic function to the aggregate proportion correct (orange lines). Baseline sensitivity was calculated with the same approach, but only including trials in which the stimulus occurred >0.1 s before or 0.5 s after a microsaccade. Shaded regions around the lines show 95% confidence intervals computed from 1,000 bootstraps. *B*: how suppression and facilitation develop over time at different spatial frequencies. The log ratio between mean sensitivity and baseline sensitivity is displayed. Warm colors show facilitation relative to baseline, and cool colors denote suppression of sensitivity. *C*: contrast sensitivity functions for 0.15-s time windows centered on the suppression (*left*) and facilitation (*right*) periods. The respective baselines are matched for subject contribution. Error bars show SE from 1,000 bootstraps.

**Fig. 3.**
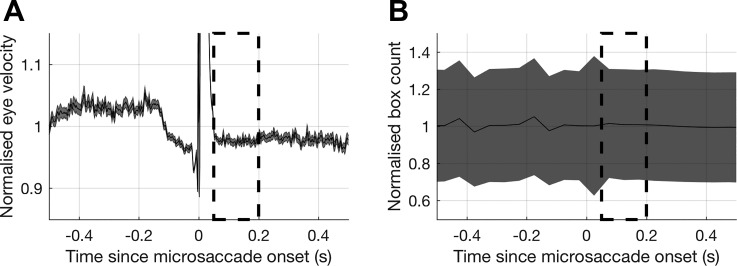
Two measures of drift as a function of time since microsaccade onset. *A*: radial eye velocity normalized to the mean radial eye velocity during the baseline window (computed from eye positions >0.1 s before a microsaccade or >0.5 s after). *B*: drift magnitude, characterized with a box count procedure, normalized to the box count during the baseline window. Gray shaded areas show SE, and dashed boxes show the facilitation time window used in Fig. 2*C* (0.05–0.2 s after a microsaccade).

Eye movements were recorded binocularly (500 Hz) with an EyeLink 1000 infrared eye tracker (SR Research, Oakville, ON, Canada). Raw gaze positions were converted to degrees of visual angle using the data from a nine-point calibration at the beginning of each block. To reduce blink artifacts, subjects were encouraged to restrict blinking to the period between a stimulus occurring and their response. Data during blink periods (pupil size = 0), along with a buffer of samples 200 ms before and after, were removed for subsequent analyses. Furthermore, trials in which a blink occurred within 100 ms of the stimulus were discarded from the analysis presented in Figs. 2 and 3. This accounted for a median of 4.5% of trials (range 0.2–38%).

Microsaccades were detected with an established velocity-threshold algorithm (Engbert and Kliegl 2003; Engbert and Mergenthaler 2006), using a threshold of 6 times the standard deviation of the median velocity. Identified saccades with duration <6 ms or amplitude <3 or >60 arcmin were discarded. Saccades within 50 ms of each other were merged to deal with situations in which overshoots were classified as separate saccades. To improve the robustness of saccade classification, microsaccades were required to overlap in time across both eyes. We verified that fixational saccades followed the main sequence (Zuber et al. 1965) by plotting amplitude against peak velocity (Fig. 1*C*). For all saccades across the population *R* was equal to 0.94, ranging from 0.86 to 0.98 across individuals. In total, 18,001 trials were analyzed.

Drift was measured with two approaches. Left eye position data for the 77,224 microsaccades in our data were collated in an epoch around microsaccade onset (1 s before to 1 s after). Position data during microsaccades were then removed from further processing. Radial eye velocity was computed from horizontal and vertical velocity and smoothed with a five-point filter. Drift magnitude was calculated with a box-counting procedure (Engbert and Mergenthaler 2006). The number of 0.01° square boxes necessary to cover the trajectory of the left eye was computed in 50-ms time bins relative to microsaccade onset. To compare drift at the time of a microsaccade with drift in the absence of a microsaccade, radial velocity and box count were normalized by their respective baseline mean, calculated from eye positions >0.1 s before microsaccade onset and >0.5 s after microsaccade onset.

#### Threshold computation and statistical comparison of groups.

For each trial, we calculated the time difference between stimulus onset and the onset of the nearest microsaccade. This single metric was then used to group trials into the time bins specified in each figure. Trials within a given temporal bin were used to form a psychometric function relating the proportion of correct responses to stimulus contrast (*c*), which was fitted by maximum likelihood with a logistic function of the form

$$p\left(\text{correct}\right)=0.5+\frac{0.5\left(1-\text{λ}\right)}{1+e^{\frac{\left(\text{μ}-c\right)}{s}}}$$

where μ is the contrast detection threshold (corresponding to 75% correct performance), λ is the lapse rate, and *s* is the slope of the psychometric function.

We initially fitted the whole data set using a single lapse rate parameter λ but allowing μ and *s* to vary across spatial frequency. The best-fitting lapse rate (λ = 0.055) and slope parameters (*s*_0.1_ = 0.105, *s*_0.33_ = 0.072, *s*_1_ = 0.094, *s*_2_ = 0.089, *s*_5_ = 0.144) were then fixed during fitting of the time-binned data, with only the threshold (μ) allowed to vary.

Ninety-five percent confidence intervals were calculated with nonparametric bootstrapping, resampling across trials before binning (1,000 repeats). Nonparametric permutation tests were performed to statistically compare across groups of thresholds (1,000 repeats). For statistical tests, to control for individual differences in microsaccade rate and sensitivity, the proportion of trials that each subject contributed to the baseline was matched for each time bin and at each spatial frequency. Thresholds were computed as the mean of 50 permutations of subject-matched trials in the calculation of both bootstraps and permutation tests.

#### Normative gain model.

To investigate whether the suppressive and facilitative effects of microsaccades on contrast sensitivity could be linked by a common mechanism, we implemented a simple model of gain control between spatial frequency channels (the MATLAB implementation of which is available at https://mfr.osf.io/render?url=https%3A%2F%2Fosf.io%2Ffbgwm%2Fdownload). The input to the model was a smoothed approximation of the variation in suppression across time and spatial frequency obtained from curve fits to the empirical data. For simplicity, we assumed that the effects of time and spatial frequency on suppression were separable.

To summarize the relative magnitude of suppression across time, we first calculated log sensitivity ratios for each time bin *t* in the lowest spatial frequency condition (sf *=* 0.1 cycles/°):

$${\text{SR}}_{t,\text{sf}=0.1}=\text{log}\left(\frac{{\text{μ}}_{t,\text{sf}=0.1}}{{\text{μ}}_{\text{baseline},\text{sf}=0.1}}\right)$$

These values were then scaled to be between 0 and 1:

$${\text{RelSupp}}_{t,\text{sf}=0.1}=\left[\frac{\text{max}({\text{SR}}_{t,\text{sf}=0.1})-{\text{SR}}_{t,\text{sf}=0.1}}{\text{max}({\text{SR}}_{t,\text{sf}=0.1})}\right]$$

and fitted with a Gaussian function of the form

$$\text{RelSupp}\_{\text{fit}}_{t,\text{sf}=0.1}=ae^{\left[{\left(\frac{t-t_{\text{peak}}}{c}\right)}^{2}\right]}$$

where *a* is the scaled log sensitivity ratio at the point of maximum suppression (set to 1), *t*_peak_ is the time bin in which maximum suppression occurs (0.02 s), and *c* is proportional to the duration of suppression (0.067 s).

To summarize the magnitude of suppression at each spatial frequency SR_sf_, we first calculated log sensitivity ratios obtained at *t*_peak_ for each spatial frequency:

$${\text{SR}}_{\text{sf},t=t_{\text{peak}}}=\text{log}\left(\frac{{\text{μ}}_{\text{sf},t=t_{\text{peak}}}}{{\text{μ}}_{\text{baseline},t=t_{\text{peak}}}}\right)$$

These values were then fitted with a third-order polynomial of the form

$${\text{SR\_fit}}_{\text{sf},t=t_{\text{peak}}}=p_{1}{\text{sf}}^{3}+p_{2}{\text{sf}}^{2}+p_{3}\text{sf}+p_{4}$$

with the best-fitting coefficients [*p*_1_ = −0.0723, *p*_2_ = −0.09754, *p*_3_ = 0.07958, *p*_4_ = −0.0641].

A smooth two-dimensional (2D) suppression profile across time and spatial frequency was then obtained by multiplying the two fitted functions together:

$${\text{SRfit}}_{t,\text{sf}}={\text{RelSupp\_fit}}_{t,\text{sf}=0.1}\times {\text{SR\_fit}}_{\text{sf},t=t_{\text{peak}}}$$

Values in SRfit*_t_*_,sf_ represent log sensitivity ratios, such that a value of zero indicates baseline performance and increasingly negative values indicate stronger suppression. A depiction of this 2D suppression profile is shown in Fig. 5*B*, along with the two separable 1D profiles over time and spatial frequency.

The model assumes that gain control mechanisms operating across spatial frequency channels function to counteract microsaccade-induced suppression. This is accomplished via divisive inhibition—the log sensitivity ratio at each spatial frequency at time *t* is divided by the mean log sensitivity ratio across spatial frequencies at a preceding time point:

$${\text{SR\_model}}_{t,\text{sf}}=\frac{{\text{SR\_model}}_{t,\text{sf}}}{\sum_{i=1}^{n}{\text{SR\_model}}_{t-t_{\text{delay}},{\text{sf}}_{i}}/n}$$

In this formulation, *t*_delay_ represents the time required for sensitivity levels to be pooled across spatial frequency channels and for the divisive inhibition to take effect.

SRfit*_t_*_,sf_ acted as the initial activity state of the model. We then iterated through each time bin in turn, dividing the sensitivity ratio in that time bin by the appropriate normalization term. The state of the model was continuously updated after each iteration, such that SRfit*_t_*_,sf_ values were progressively replaced by SR_model*_t_*_,sf_ values until all iterations were complete. This process led to attenuation of the initial suppression in the model output (SR_model*_t_*_,sf_). To maintain correspondence between the model and empirical data set, we ran the model through once and then used the ratio of the SR values at peak suppression in the original fit and the model output to scale parameter *a* from RelSupp_fit*_t_*_,sf = 0.1_.

$$a=\left(\frac{{\text{SRfit}}_{t=t_{\text{peak}},\text{sf}=0.1}}{{\text{SR\_model}}_{t=t_{\text{peak}},\text{sf}=0.1}}\right)$$

We then ran the model again but with RelSupp_fit*_t_*_,sf = 0.1_ computed with the new value of *a* (which was scaled to [2.25,1.38,1.29,1.01] for the four values of *t*_delay_ displayed in Fig. 5*C* ([0,0.04,0.06,0.14]). Across different simulations, the value of *t*_delay_ was the only free parameter manipulated.

## RESULTS

### 

#### Sensitivity changes due to microsaccades.

We recorded subjects’ fixational eye movements as they performed a contrast detection task for stimuli with different spatial frequencies (materials and methods, Fig. 1*A*). As they were performing the task, subjects unconsciously made between 0.3 and 2.3 microsaccades per second. Subjects could not predict when a stimulus would appear, and microsaccades were randomly distributed around stimulus onset, at least for the time window used in subsequent analyses (Fig. 1*B*). While microsaccade rate is inhibited by stimulus appearance (Engbert and Kliegl 2003), most stimuli were presented at low contrast around threshold, leading to only a weak microsaccadic inhibition (see Bonneh et al. 2015; Scholes et al. 2015; White and Rolfs 2016). Microsaccades displayed the relationship between peak velocity and amplitude characteristic of ballistic eye movements (Fig. 1*C*) and were predominantly oriented horizontally (Fig. 1*D*).

Contrast detection thresholds were computed relative to microsaccade onset by binning trials that fell within a given time interval and fitting a logistic function to the aggregate proportion correct for those binned trials, using a maximum likelihood criterion (see materials and methods). Figure 2*A* displays contrast sensitivity (1/threshold) relative to microsaccade onset for each spatial frequency. Baseline sensitivity, calculated from trials in which there was no microsaccade, is also displayed. In line with previous work on large volitional saccades (Diamond et al. 2000; Knöll et al. 2011) and microsaccades (Chen and Hafed 2017; Hass and Horwitz 2011), visual sensitivity was lower around the time that the eye was moving. Although suppression was evident at all of the spatial frequencies tested, the effect was largest for low-spatial frequency stimuli. Unexpectedly, for higher spatial frequencies, perisaccadic suppression was followed by an increase in sensitivity ∼100–200 ms after microsaccade onset. Differences in the tuning of perisaccadic suppression and postsaccadic facilitation led to a dynamic fluctuation in spatial sensitivity as a function of the time since microsaccade onset (shown in the log sensitivity ratio, relative to baseline sensitivity, in Fig. 2*B*). Figure 2*C* shows contrast sensitivity functions calculated within two 0.15-s time windows that putatively represent the suppression and facilitation periods. Baseline contrast sensitivity functions are also shown. To ensure that changes in sensitivity were not due to variations in the relative influence of individual subjects, the proportion of trials contributed by each subject in each time bin was matched in its associated baseline. Suppression was greatest for low spatial frequencies and decreased in magnitude as spatial frequency increased, though significant suppression was present for all spatial frequencies (*P* < 0.01, nonparametric permutation test). In contrast, facilitation was greatest at 2 cycles/° and was significant for spatial frequencies at and above 1 cycle/° (*P* < 0.01, nonparametric permutation test).

#### Is facilitation due to postmicrosaccadic eye movements?

An interesting aspect of the facilitation reported here is that it occurs some time after the eye has completed the microsaccade, during a period of slow drift. Movement of the visual scene across the retina by slow drift causes a change in spatiotemporal tuning that leads to facilitation of high-spatial frequency stimuli but not low-spatial frequency stimuli (Boi et al. 2017; Rucci et al. 2007). Thus if drift were responsible for the facilitation noted here then we would expect the largest effects to be observed at the highest spatial frequency (5 cycles/°); however, this was not the case.

It is unlikely that drift is responsible for the facilitation, but to reinforce this we examined eye movements as a function of the time since microsaccade onset for all of the microsaccades in our data. If facilitation were due to a change in drift characteristics then we might expect to see a change in drift velocity or magnitude during the facilitation period. Figure 3 shows two approaches to characterize drift as a function of time since microsaccade onset (after removal of microsaccade events from the data; see materials and methods). Irrespective of the way it is measured, drift is relatively constant from ∼0.05 s after microsaccade onset: there is no change in drift velocity/magnitude that could account for the period of facilitation that we observe (the 0.05–0.2 s window used in Fig. 2*C*). This is true when drift is averaged both relative to the full set of microsaccades displayed in Fig. 3 and relative to microsaccades partitioned based on the spatial frequency of the stimulus (not shown).

#### Contribution of smear to microsaccadic suppression.

**Fig. 4.**
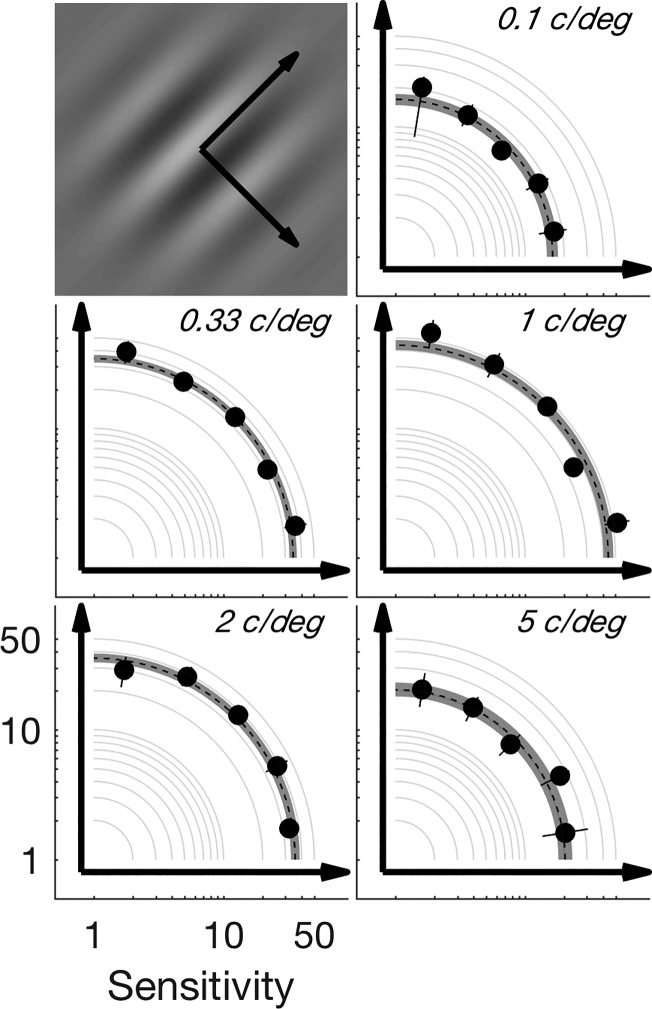
Sensitivity as a function of the orientation difference between the stimulus and microsaccade for each spatial frequency, using the same suppression time bin as in Fig. 2*C* (0.1 s before to 0.05 s after a microsaccade). Trials were partitioned depending on the angle between the microsaccade and stimulus (arrows in *top left* panel). The points in each panel represent the center of a bin with 18° width, with the leftmost (rightmost) point showing sensitivity for microsaccades parallel (orthogonal) to the stimulus (black arrows in each panel show how the axes are translated from *top left* panel). Solid black lines indicate 95% confidence intervals (CIs). Sensitivity for all orientations (orange points in Fig. 2*C*, *left*) is displayed as a dashed black line, with 95% CIs indicated by gray shading.

In studies of saccadic suppression (Burr et al. 1994; Diamond et al. 2000; Knöll et al. 2011), smear of the stimulus across the retina could be somewhat controlled for by directing the (voluntary) saccade parallel to the orientation of the stimulus. In general, microsaccades occur without the explicit knowledge of the subject, and so microsaccade orientation could not be predicted in advance. Thus it is possible that some of the suppression that we report is due to smear. However, smear should exert its greatest effect at higher spatial frequencies, where we, in fact, observe the least suppression. Similarly, smear-induced suppression should be greatest for those trials in which the microsaccade was orthogonal to the orientation of the grating. To test this, we grouped trials depending on the difference between microsaccade and stimulus orientation, ranging from 0° (microsaccade parallel to grating) to 90° (microsaccade orthogonal to grating). Figure 4 shows sensitivity estimates obtained by partitioning perisaccadic trials according to the difference in microsaccade and stimulus orientation. In contrast to the predictions of a retinal smear mechanism, sensitivity does not vary systematically with the direction of the microsaccade relative to the stimulus waveform, across the spatial frequencies tested.

#### Mutual inhibition model of microsaccadic suppression and facilitation.

Suppression and facilitation of sensitivity may be caused by independent processes; however, their proximity in time and complementary tuning suggest a common underlying process. Saccadic suppression has previously been described as a dynamic reduction of divisive gain (Burr and Morrone 1996; Knöll et al. 2011) and, more recently, as a reweighting of sensory information in a Bayesian estimator framework (Crevecoeur and Kording 2017). Our data are consistent with both of these approaches, at least during the period of microsaccadic suppression. While either framework could be extended to account for postsaccadic facilitation of sensitivity, it is unclear how they would account for the different spatial frequency tuning of suppression and facilitation reported here.

We propose that facilitation could arise as a result of microsaccadic suppression causing an imbalance in time-dependent gain control mechanisms between spatial frequency channels in the visual system (Blakemore and Campbell 1969). As a proof of concept, we implemented a model in which the sensitivity at each spatial frequency and time point is normalized by the mean sensitivity across spatial frequency from a preceding time point. The only free parameter in the model, *t*_delay_, represents the time required for sensitivity levels to be pooled across spatial frequency channels and for this divisive inhibition to take effect.

**Fig. 5.**
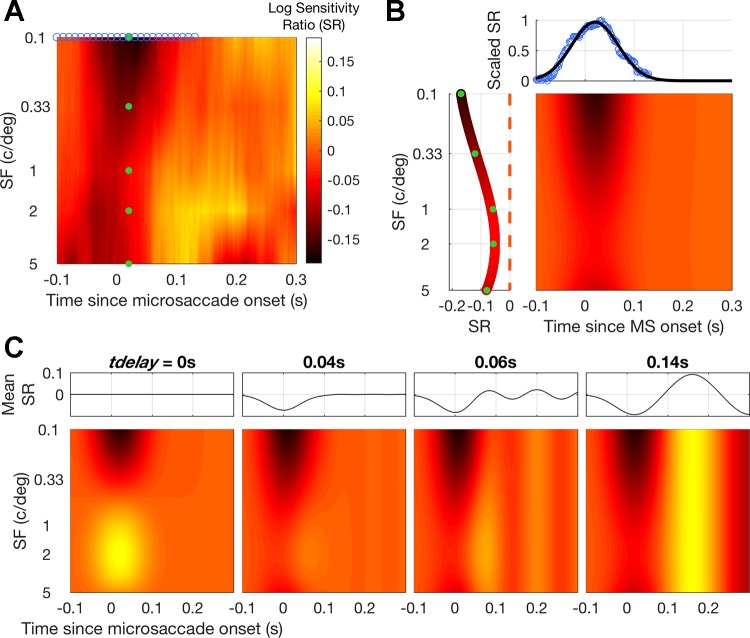
Delayed normative gain model of microsaccadic suppression and facilitation. *A*: a smooth 2D suppression profile was simulated using the empirical data (shown here) by multiplying 1D fits to the log sensitivity ratio (SR) across time and spatial frequency. *B*: specifically, the SR from the time bin with peak suppression (*t* = 0.02 s, indicated by green dots here and in *A*) was fit with a 3rd-order polynomial (*left*), and the SR for the lowest spatial frequency condition (0.01 cycle/°, indicated by blue circles here and in *A*) was scaled and fit with a Gaussian (*top*). The resultant 2D profile is shown in the main panel. *C*: the suppression profile acted as the initial state of the model. Each time bin was iterated through, in turn, and the current SR values were divided by the mean SR from a time bin *t*_delay_ seconds back in time, with model SR continuously updated. *Bottom*: the final model SR as *t*_delay_ was varied (indicated above each panel). *Top*: the mean SR.

The model is a 2D representation of sensitivity over time and spatial frequency (analogous to the log sensitivity ratio shown in Fig. 2*B* and redrawn in Fig. 5*A*). Initially, microsaccadic suppression was simulated (main panel, Fig. 5*B*) by multiplying a curve fit to the sensitivity ratio across time with a curve fit to the sensitivity ratio across spatial frequency (Fig. 5, *A* and *B*). This smooth 2D suppression profile acted as the initial state of the model. Figure 5*C* demonstrates how facilitation can occur as the result of divisive normalization and how the spatial tuning and timing of this facilitation vary as a function of *t*_delay_. With no delay (*t*_delay_ = 0; Fig. 5*C*, *left*), normalization acted to balance suppression and facilitation at each time point such that the net sensitivity ratio across spatial frequency was 0 (shown in Fig. 5*C*, *top* *left*). As the delay was increased, facilitation moved forward in time relative to microsaccade onset (with the tuning and timing of sensitivity changes becoming more like those in the experimental data). For longer delays (e.g., 0.14 s and above), the timing of the facilitation increased as a function of the delay, but the tuning of the facilitation became less specific across spatial frequency.

## DISCUSSION

Much of the work that describes changes in luminance contrast sensitivity during large saccades has focused on the suppression of sensitivity before and during the eye movement. We have demonstrated that for microsaccades suppression is part of a dynamic variation in spatial sensitivity that evolves as a function of time since microsaccade onset. Suppression occurs before and during microsaccades and is strongest at low spatial frequencies, while facilitation occurs ∼100–200 ms after microsaccade onset and is strongest at peak spatial frequencies. Current models of changes in sensitivity around the time of saccades focus on suppressive effects. We demonstrated how a time-dependent normative gain of sensitivity could account for the spatially tuned facilitation that occurs after a microsaccade.

### 

#### Postmicrosaccadic facilitation of sensitivity.

The majority of reports of postsaccadic stimulus detection enhancement have been restricted to chromatic stimuli. Sensitivity is increased ∼100 ms after saccade onset (Burr et al. 1994; Diamond et al. 2000; Knöll et al. 2011). This facilitation is likely to be mediated by different mechanisms to saccadic suppression because postsaccadic enhancement is still present with saccadelike motion of the whole stimulus display while saccadic suppression is not (Diamond et al. 2000). For monochromatic stimuli, one study has demonstrated postsaccadic enhancement of detection thresholds (Burr et al. 1982), but only under conditions of very low luminance; suppressive effects dominated for higher luminance levels comparable to those used here. Several physiological studies (Ibbotson et al. 2008; Leopold and Logothetis 1998; Rajkai et al. 2008; Reppas et al. 2002; Royal et al. 2006) have demonstrated postsaccadic increases in neural activity at around the same time that we observed facilitation, although none has investigated the spatial tuning of this neural enhancement. Similarly, facilitation of neural responses has also been demonstrated after microsaccades (Bellet et al. 2017) for a spatial frequency (2.2 cycles/°) that is similar to the spatial frequencies for which we report increased sensitivity. Facilitation of discrimination sensitivity at the end point of saccades has been demonstrated (Dorr and Bex 2013; Rolfs et al. 2011). However, this is proposed to be due to predictive remapping of attention (Cavanagh et al. 2010; Rolfs et al. 2011) and is thus unlikely to depend on the spatial content of the stimulus. Consistent with this, Dorr and Bex (2013) reported facilitation for both low- and high-frequency stimuli. Finally, sensitivity for chromatic and very high-spatial frequency stimuli (12 cycles/°) is facilitated during smooth pursuit, but unlike in the present study, critically this occurs before the eyes start to move (Schütz et al. 2008).

A recent study by Bellet et al. (2017) demonstrated a postmicrosaccadic increase in performance in a task in which subjects detected the presence of a small dot stimulus. Interestingly, increases in performance (in a time window of 50–400 ms after microsaccade onset) were predominantly in the hemifield into which a microsaccade was directed. In a second task, Bellet et al. (2017) also demonstrated postmicrosaccadic oscillations of saccadic reaction times to a suprathreshold dot stimulus that were initially present in the hemifield into which a microsaccade was directed before switching to the opposite hemifield. An interesting avenue for future work is to examine how these spatial features of facilitation interact with the dependence on spatial frequency demonstrated in our study.

#### Microsaccadic suppression.

The timing and spatial tuning of microsaccadic suppression are similar to those observed for large saccades, albeit with a smaller magnitude. The smaller magnitude of suppression is not surprising given that suppression magnitude has previously been shown to scale with the size of voluntary saccades (Ridder and Tomlinson 1997). We employed large stimuli (standard deviation of 5**°**), so the suppression and facilitation that we have shown are not limited to the fovea. It may be that a more spatially constrained investigation of sensitivity within the fovea would demonstrate a greater degree of suppression (see, e.g., Rucci and Mostofi 2017). Microsaccadic suppression began up to 50 ms before the eyes began to move, in line with previous reports for large, volitional saccades (Diamond et al. 2000; Knöll et al. 2011). In many areas of the brain, neural responses are attenuated before a saccade is initiated. Presaccadic suppression of spiking activity begins ~50–100 ms before the eye starts to move in lateral geniculate nucleus (Reppas et al. 2002; Royal et al. 2006), V1 (Kagan et al. 2008), and middle temporal/medial superior temporal (Bremmer et al. 2009; Ibbotson et al. 2008) and lateral/ventral intraparietal (Bremmer et al. 2009) areas. Suppression of neural responses in SC operates over a similar time course before microsaccades, as it does before saccades in other brain areas (Hafed and Krauzlis 2010). Microsaccadic suppression was greatest for low spatial frequencies, as previously found for large saccades (Burr et al. 1994; Volkmann et al. 1978), and for delays in saccadic reaction times (Chen and Hafed 2017). The similarity between micro- and macrosaccadic suppression is perhaps unsurprising given that the current weight of evidence suggests that microsaccades and saccades are members of an oculomotor continuum generated by the same neural circuitry in the SC (Hafed et al. 2009; Hafed and Krauzlis 2012).

#### Mechanism to account for suppression and facilitation.

The differences in spatial tuning between suppression and facilitation constrain possible underlying mechanisms. Our analysis of eye movements occurring during the facilitation period demonstrated that there were no discernible differences in drift magnitude or velocity relative to baseline. We demonstrated how facilitation could result from delayed normative gain across spatial frequency channels, in which facilitation arises from the imbalance in gain caused by suppression. The presence of spatially tuned channels in the visual system is uncontroversial (Blakemore and Campbell 1969); however, the degree of independence of these channels is still a matter for debate. Early psychophysical adaptation studies demonstrated facilitation of spatial frequencies far from the adapting frequency (De Valois 1977; Tolhurst and Barfield 1978). Interestingly, the facilitative effects were reported to be strongest at spatial frequencies ∼2–3 octaves higher than the adapting spatial frequency, in general agreement with the spatial frequency difference between suppression and facilitation in our data.

Our goal was to demonstrate that a normative gain model could qualitatively account for our data. A challenge to developing a quantitative account would be to incorporate features that are likely to be important physiologically, such as the bandwidth and time course of inhibitory connections across spatial frequency channels. The timing of facilitation revealed here is in general agreement with estimates of the time constant of divisive contrast gain control revealed by psychophysical studies (100–200 ms: Geisler and Albrecht 1992; Wilson 1993; Wilson and Kim 1998). Given the similarity between micro- and macrosaccade generation and features, one would expect that microsaccadic facilitation would generalize to larger saccades. If this is true, then saccade paradigms could potentially provide an indirect means to explore the interdependence between spatial frequency channels.

Our data contribute to a growing body of evidence suggesting that visual sensitivity is far from constant. Recent evidence suggests that suppression and facilitation around the time of saccades may be part of an ongoing oscillation of sensitivity that can be reset by a motor action, such as a saccade (Bellet et al. 2017; Benedetto and Morrone 2017; Tomassini et al. 2015). If these oscillations were due to phase-resetting induced by a motor act, it is unclear why one would observe spatial tuning of either suppression or facilitation, as revealed by our data. We extended the time window of analysis for our data and found no evidence for premicrosaccadic oscillations of sensitivity (as was described for larger saccades in Benedetto and Morrone 2017). Postmicrosaccadic sensitivity fluctuated around baseline but without obvious oscillatory behavior (consistent with the findings of Bellet et al. 2017). A physiological mechanism for the oscillation in visual sensitivity is yet to be proposed. Interestingly, an emergent property of our normative gain model is a weaker, broadly tuned oscillation of sensitivity after the initial period of facilitation. Our model could thus provide a framework that can account for both suppression and facilitation around the time of eye movements and oscillations of sensitivity.

Whether postmicrosaccadic facilitation has any bearing on everyday vision is open to question. Anecdotally, patients undergoing clinical testing of contrast sensitivity (e.g., Pelli-Robson CS chart; Pelli et al. 1988) often report that making small eye movements and waiting is an effective strategy for revealing the presence of letter stimuli close to their detection threshold. It may be the case that in this situation the patient is attempting to exploit the changes in sensitivity that occur after small changes in eye position. Similarly, our data provide a parsimonious explanation for the discrepancy between early and recent studies highlighted in introduction. Mostofi et al. (2016) reported that sensitivity was higher in trials that included one or more large microsaccades compared with trials that included only drift, for stimuli with a spatial frequency at which facilitation likely occurs. Critically, Mostofi et al. used contrast-ramped stimuli of 1-s duration (full contrast for 0.5 s), thus allowing subjects to exploit a strategy similar to the patients mentioned above. The dynamic nature of sensitivity that we have demonstrated suggests that precise measures of contrast threshold require brief exposure times.

## GRANTS

This work was supported by the Wellcome Trust [Grant WT097387].

## DISCLOSURES

 No conflicts of interest, financial or otherwise, are declared by the authors.

## AUTHOR CONTRIBUTIONS

C.S., P.V.M., and N.W.R. conceived and designed research; C.S. performed experiments; C.S. analyzed data; C.S. interpreted results of experiments; C.S. prepared figures; C.S. drafted manuscript; C.S., P.V.M., and N.W.R. edited and revised manuscript; C.S., P.V.M., and N.W.R. approved final version of manuscript.
